# Two case reports of fetal alcohol syndrome: broadening into the spectrum of cardiac disease to personalize and to improve clinical assessment

**DOI:** 10.1186/s13052-019-0759-y

**Published:** 2019-12-19

**Authors:** R. Onesimo, C. De Rose, A. B. Delogu, A. Battista, C. Leoni, S. Veltri, G. De Rosa, G. Zampino

**Affiliations:** 1 Center for Rare Diseases and Birth Defects, Department of Woman and Child Health, Fondazione Policlinico Universitario A. Gemelli IRCCS, Largo Francesco Vito 8, 00168 Rome, IT Italy; 2 Pediatric Cardiologic Unit, Department of Woman and Child Health, Fondazione Policlinico Universitario A. Gemelli IRCCS, Rome, Italy; 3 Università Cattolica del Sacro Cuore, Institute of Pediatrics, Rome, Italy

**Keywords:** FASD, Fetal alcohol syndrome, Disability, Cardiac defect, Arrhythmia

## Abstract

**Background:**

Fetal alcohol spectrum disorder (FASD) refers to a broad spectrum of disabilities, in infants and children, resulting from moderate to excessive prenatal alcohol exposure.

Significant associations with alcohol exposure were already reported with congenital structural heart defects: i.e. ventricular septal defects, atrial septal defects, conotruncal defects.

**Cases presentation:**

We describe two cases of children with FASD, both admitted to the Center for Rare Diseases and Birth Defects of Policlinico Universitario Agostino Gemelli, in whom asymptomatic cardiac rhythm alterations were detected in absence of structural cardiovascular system anomalies or cardiac channelopathies.

**Conclusions:**

No other reports about cardiac rhythm anomalies in individuals affected by FASD are actually available from the literature.

We would like to make an alert for clinician, given the possibility of finding anomalies of heart conduction and rhythm in children affected by FASD even without structural congenital heart disease.

## Introduction

The term fetal alcohol spectrum disorder (FASD) refers to a broad spectrum of disabilities, in infants and children, resulting from moderate to excessive prenatal alcohol exposure [1]. It includes fetal alcohol syndrome (FAS), partial fetal alcohol syndrome (pFAS), Alcohol Related Birth Defects (ARBD) and Alcohol Related Neurodevelopmental Disorders (ARND) [2].

FAS is characterized by the presence of all of the following criteria: two of the three typical facial features (short palpebral fissures, thin vermillion border and a smooth philtrum), growth retardation and central nervous system (CNS) anomalies. FAS diagnosis does not require documentation of prenatal alcohol exposure. pFAS is defined by two of the previously reported facial features plus other structural defects. ARBD has physical defects secondary to known fetal alcohol exposure, without neurobehavioral disorders. ARND refers to neuropsychiatric impairment caused by prenatal alcohol exposure in the absence of physical defects [3, 4].

According to recent US data, the prevalence of FASD vary considerably from 24 to 48 per 1000 children; however, if we consider particular subpopulation groups, FASD rates might be considerably higher. FASD incidence in Europe is estimated to be 1–3:10000; in the US 2–7:1000 and 2–5% respectively for FAS and FASD [5].

In Lazio (Italy) prevalence of FAS is 3.7–7.4 per 1000 children, the rate of FASD is 20.3 to 40.5 per 1000 and estimated between 2.3 and 4.1% of all children [6].

FASD is the most common cause of mental retardation acquired during childhood; however, structural involvement of other systems (cardiovascular, renal, musculoskeletal, ocular and auditory systems) has already been associated with this condition [7].

Significant associations with alcohol exposure were reported with ventricular septal defects and atrial septal defects. Furthermore, mothers who drink alcohol during pregnancy have 1.64 fold times increased risk to have a newborn affected by conotruncal defects (CTDs) subtypes such as transposition of the Great Arteries (dTGA) [7]. This evidence suggests both prenatal heavy drinking and binge drinking are strongly associated with an overall increased risk to present babies with congenital heart defects [7].

We know that alcohol consumption during pregnancy can have negative effect on the fetus cardiovascular system; however only congenital structural heart defects have been reported to date [7]. No reports about cardiac rhythm anomalies in individuals affected by FASD are actually available from the literature.

Herein we report the cases of two children with FASD, both admitted to the Center for Rare Diseases and Birth Defects of our Institution, in whom asymptomatic cardiac rhythm alterations were detected in absence of structural cardiovascular system anomalies during routine follow-up.

### Case 1

The proband was admitted for the first time at 7 year old, to confirm the hypothesis of FASD. The child was born in Russia and he was adopted at the age of four. Few data were available from his perinatal history: he was born at term, with detected microcephaly. His mother was an alcoholic; however, no further information were available about the alcohol rate during pregnancy.

At the time of the adoption, the child had psychomotor delay with poor language development; retinal angiopathy; the echocardiography evaluation showed a normal heart anatomy.

In Italy, he underwent neuropsychological tests confirming a mild intellectual disability. Genetic consult was performed and chromosomal rearrangements were absent at arrayCGH.

In the presence of neurological impairment, growth delay and typical facial features (short palpebral fissures, thin vermillion border, and a smooth philtrum), after multidisciplinary evaluations, diagnosis of FAS was confirmed by exclusion of the other conditions.

At the last clinical evaluation, performed at the age of 8 years, he showed a global good clinical setting. According to the CDC growth charts, height and weight were within the normal limits, cranial circumference was − 1.8 SD. Child neurologist confirmed behavioral disorders and mild intellectual disability.

On physical examination, the heart rate, blood pressure and respiratory rate were normal, but cardiac auscultation revealed an irregular rhythm, so that an ECG with a long rhythm strip was required. On ECG, premature ventricular contractions (PVCs) were identified; repeated echo study excluded structural heart disease (except for a patent foramen ovale) or functional abnormalities.

On 24-h Holter monitoring, PVCs were frequent (14,567/24 h) and appeared uniform and isolated with ventricular bigeminy and trigeminy; no couplets, triplets or ventricular tachycardia were detected.

On exercise stress testing, rare monomorphic PVCs were present, without symptoms at rest or during exercise. PVCs demonstrated a right bundle branch block morphology with a right axis deviation, suggesting a fascicular origin.

Laboratory studies including full blood count (for anemia), electrolytes, blood glucose, and thyroid function testing were performed and were normal.

Since, the patient was asymptomatic - without underlying heart disease - and he had uniform and isolated PVCs - without induction or exacerbation of arrhythmia with exercise - further extensive investigation or treatment was not considered to be required and cardiac follow up with ECG, 24-h Holter monitoring and exercise stress testing was scheduled.

### Case 2

A 8 year old male was admitted to our Center to confirm a diagnosis of FASD.

The child lived in Poland until he was 6 years and a history of maternal alcohol abuse during pregnancy was recorded from past medical history reports. He had no history of growth delay, microcephaly or cardiac abnormalities. Adopted parents reported generic emotional and behavioral problems.

Clinical evaluation showed the typical phenotype of FASD: telecantus, jaw hypoplasia, nasal saddle hypoplasia and thin vermillion border.

A neuropsychiatric evaluation and a cognitive assessment (Leiter scale) were performed, with no evidence of major problems with the exception for low attention level. Array CGH did not reveal anomalies and genetic consultation confirmed FASD.

Six months after the first physical examination, the child reported complaints of palpitations, with sudden start and stop of rapid heartbeats, without other signs or symptoms. Then a complete cardiac check was performed.

An echocardiographic evaluation detected a structurally normal heart (except for a small patent foramen ovale) without functional abnormalities. Routine ECG during office visit revealed sinus rhythm with frequent premature atrial contractions (PACs). On 24-h Holter monitoring, sinus rhythm was interrupted by frequent PACs and short runs of ectopic atrial tachycardia (maximal heart rate 146 beats/minute, 6 beats).

On exercise stress testing, many isolated premature atrial contractions were present, with occasional couplets and triplets and frequent runs of atrial ectopic tachycardia (the longer supraventricular tachycardia (SVT) with 12 beats at maximal heart rate 160 beats/minute); at high workload, isolated and monomorphic ectopic ventricular beats appeared, with a left bundle branch block QRS morphology; no ventricular repolarization abnormalities were detected.

To rule out other medical conditions suspected as a cause of supraventricular tachycardia, laboratory studies including full blood count (for anemia), electrolytes, blood glucose, and thyroid function testing were performed and were normal.

Since, no heart disease was found and the patient tolerated very well this kind of SVT - due to short runs of tachycardia at low heart rate, without any hemodynamic significance - specific treatment was not indicated and cardiac follow up with ECG, 24-h Holter monitoring and exercise stress testing was scheduled.

## Discussion

A different alcohol rate abuse during pregnancy causes a broad spectrum of disorders known as FASD, in which FAS who includes the presence of peculiar facial features, growth delay and CNS anomalies represent the worst condition [3].

Due to the lack of genetic or biochemical diagnostic tests, the decisive step to identify a patient with FASD is to ascertain maternal alcohol consumption during pregnancy. The absence of this data, however, does not exclude the diagnosis that must be formalized following the recent guidelines [Hoyme HE, 2016] which are based on the multidisciplinary approach to the mother-child dyad and are aimed at analyzing three essential aspects of the syndrome: - the morphological anonymities of the newborn; − the child’s neuropsychological, intellectual and social development; − maternal risk factors [3].

Structural defects in cardiovascular, renal, musculoskeletal, ocular, and auditory systems have already been described in FASD [8]. Interestingly no data about cardiac rhythm disturbances in patients with FASD have never been reported to date.

In the neonatal period, FASD may be suspected by the presence of small infant for gestational age (SGA), microcephaly and typical dysmorphic features (middle-facial hypoplasia, short palpebral fissures, elongated and flat nasolabial filter, thin upper lip, telecantus, jaw hypoplasia, the anomalies of the ears, the poorly modeled pavilions).

The evidence of alcohol abuse during pregnancy helps clinicians in the differential diagnosis with other syndromic disorders.

By passing time, neuropsychiatric issues may progressively appear such as psychomotor delay, behavioral disorders and attention deficit, scholastic and social problems.

Our patients were both diagnosed as having FASD during scholar age, no cardiac concerns were reported in the previous years.

In Case 1 the ECG was performed to confirm extrasystoles detected during routine cardiac auscultation whereas in Case 2 was performed because of reported episodes of palpitations.

The cardiac evaluation showed a sinus rhythm with isolated but frequent junctional and monomorphous ventricular extrasystoles in case 1, in absence of any other cardiac symptom. The second child (case 2) showed a sinus rhythm with rare ectopic ventricular beats, frequent ectopic supraventricular beats and episodes of supraventricular tachycardia associated to palpitations.

In both cases, in the absence of clinical history of sincope and ECG findings of QT prolongation, repolarization anomalies and/or complex ventricular arrhythmias, molecular investigations for cardiac channelopathies were not performed.

Generally, *premature ventricular contractions (PVCs)* may appear in otherwise healthy children and are benign, particularly if they are uniform and disappear or become less frequent with exercise. PVCs are more significant if they are associated with underlying heart disease as preoperative or postoperative status or cardiomyopathy, if there is a history of syncope or a family history of sudden death, if they are precipitated by activity, if they are multiform, particularly couplets, if there are runs of PVCs with symptoms and if there are incessant or frequent episodes of paroxysmal ventricular tachycardia. In patients without structural heart disease, ventricular ectopic beats are usually monomorphic, isolated, they manifest at low heart rates and disappear during exercise; this benign condition usually does not require any treatment. On the other hand, the presence of symptomatic extrasystoles, polymorphic ectopic beats, usually organized in pairs or triplets, which appear or increase with stress must be investigated in depth [9, 10].

Generally, *premature atrial contractions (PACs)* may appears in healthy children, including newborns, without any hemodynamic significance, and usually no treatment is needed. Supraventricular isolated extrasystoles can be found in many healthy children and no therapy is required. These type of extrasystoles can be followed by a normal or abnormal QRS (LBBB or RBBB shape); in some cases the input can be stopped at the level of the AV node, resulting in the absence of the following QRS (apparent pause). In newborns, atrial extrasystoles are quite frequent and usually regress within the first few weeks of life; atrial extrasystoles are a benign condition and there is no risk of degeneration in more severe arrhythmias. In older children, frequent supraventricular extrasystoles may anticipate the development of a breast node dysfunction (see sick sinus syndrome) especially following cardiac surgery or in association with cardiomyopathy [9, 10].

*Supraventricular tachycardia (SVT)* is the most common arrhythmia in infancy and usually affects subjects with structural normal heart. SVT is idiopathic in over 50% of cases, especially in infants. *Ectopic atrial tachycardia* is a type of supraventricular tachycardia (SVT) believed to be secondary to increased automaticity of nonsinus atrial focus or foci and most patients have a structurally normal heart (idiopathic). Patients with congenital heart diseases, both during natural history or after surgery, have an higher risk of developing SVT; it is estimated that the 9–32% of these patients present at list one episode of SVT. Most of the idiopathic SVTs remain asymptomatic [11]. However, in children with negative history for cardiac conditions, episodes of palpitations or tachycardia usually do not have a cardiac origin but occur as a physiological response to external or internal factors (e.g. anxiety, fever, hypovolemia, orthostatic hypotension). Moreover, these episodes might be related to extra cardiac conditions: pheochromocytoma, arteriovenous fistula, intake of stimulant drugs, anemia, hyperthyroidism, electrolyte imbalances and gastrointestinal problems (especially gastro-esophageal reflux) [12–14]. In chronic cases of ectopic atrial tachycardia, cardiac heart failure may occur, and there is a high association with tachycardia-induced cardiomyopathy, refractory to medical therapy and cardioversion. In these cases, the goal may be to slow the ventricular rate rather than to try to convert the arrhythmia to sinus rhythm [9,10].

Direct effects of alcohol dependence on the cardiovascular system and cardiac rhythm in the general population have already been reported; however the pathogenetic mechanisms related to the pro-arrhythmic effects of alcohol are still unclear. The risk of arrhythmias is dose-dependent, not influenced by preexisting cardiovascular diseases or heart failure and can affect otherwise healthy subjects. Acute alcohol toxicity can lead to cardiac contraction impairment with rhythm disturbances (holiday heart syndrome), transient ischemic attacks and, in rare cases, sudden cardiac death. Cardiac effects of chronic high alcohol consumption include ventricular dysfunction, chronic rhythm disturbances, alcoholic cardiomyopathy and coronary artery disease [15, 16].

The exact teratogenic mechanism of alcohol on fetal development is still unclear. Several factors such as the onset of alcohol abuse during pregnancy, daily dose and maternal comorbidities may influence the severity of FASD. Toxic effect on autonomic nervous system may not be excluded and could explain the presence of arrhythmic abnormalities in the absence of structural cardiac defects in our patients.

We do not know if the presence of arrhythmic abnormalities of our patients is a casual association or a feature that could enlarge the FASD spectrum. Our report is an alert for clinicians.

To define the prevalence of this event it is necessary to study systematically a wider cohort of patients with FASD.

Given the possibility of finding anomalies of heart conduction and rhythm in children affected by FASD even without congenital structural defect, we would suggest to clinicians to include periodic ECGs monitoring in the follow-up of these children, even without the presence of suggestive cardiac symptoms.

## Conclusion

FASD is not a genetic but rather a multisystem disorder, hence multidisciplinary team should perform FASD follow up. Since there is no treatment to reverse alcohol-induced damages, primary and secondary preventions are the only chances to detect and if necessary to treat heart abnormalities. Finally, on the light of the described clinical experience and related findings, a screening for arrhythmias by using ECG even in children affected by FASD without structural congenital heart disease has to be performed.

## Abbreviations

ARBD: Alcohol related birth defects
ARND: Alcohol related neurodevelopmental disorders
CNS: Central nervous system
CTDs: ConoTruncal defects
dTGA: Transposition of the great arteries
FAS: Fetal alcohol syndrome
FASd: Fetal alcohol spectrum disorder
PACs: Premature atrial contractions
pFAS: partial Fetal Alcohol Syndrome
PVCs: Premature ventricular contractions
SGA: Small for gestational age
SVT: Supraventricular tachycardia
